# First European experience with a leadless atrial pacemaker

**DOI:** 10.1016/j.hroo.2025.05.011

**Published:** 2025-05-16

**Authors:** Elia von Felten, Alexander Breitenstein, Andreas Müller, Caroline Wiederkehr, Theresa Brix, Raban Jeger, Daniel Hofer

**Affiliations:** 1 Department of Cardiology, Triemli Hospital Zürich, Zürich, Switzerland; 2 Department of Cardiology, University Heart Center, University Hospital Zürich, Zürich, Switzerland; 3 University of Basel, Basel, Switzerland

**Keywords:** Leadless pacemaker, Atrial, Helix fixation, Dual chamber pacemaker, Implant procedure, Pacemaker complications, AVEIR

## Abstract

**Background:**

Leadless pacemakers (LPs) can reduce long-term complications compared with conventional devices. However, previous studies have primarily focused on single chamber right ventricular LPs.

**Objective:**

This study aimed to evaluate the implantation, safety, and device performance characteristics in a first real-world European use of an active fixation atrial LP for either dual chamber or single chamber pacing.

**Methods:**

For this prospective, single-center, single-operator analysis, we included consecutive patients undergoing implantation of an active fixation atrial LP either as a standalone therapy or as part of a dual chamber LP system.

**Results:**

Of the 45 included patients, 22 (48.9%) underwent de novo dual chamber, 9 (20%) an upgrade from ventricular single chamber to dual chamber, and 14 (31.1%) an atrial single chamber LP implantation. All implantations were successful and without the need for device repositioning. The median procedure time was 35 minutes (interquartile range 30–40), and complications occurred in 2 patients (4.4%) who developed pericardial effusions. Sensing, impedance, and stimulation thresholds of the atrial and ventricular device remained stable or improved over a median follow-up of 21 days. Median implant-to-implant throughput was 88% for atrial-to-ventricular and 87% for ventricular-to-atrial. The estimated median battery life of the atrial device was significantly higher in single chamber vs dual chamber settings (11.9 years vs 5.8 years, *P <* .001).

**Conclusion:**

Our first real-world European experience demonstrates that leadless atrial pacemaker implantation can be performed efficiently and safely, with satisfactory device measurements during the early follow-up period.

## Graphical abstract

**Figure:**
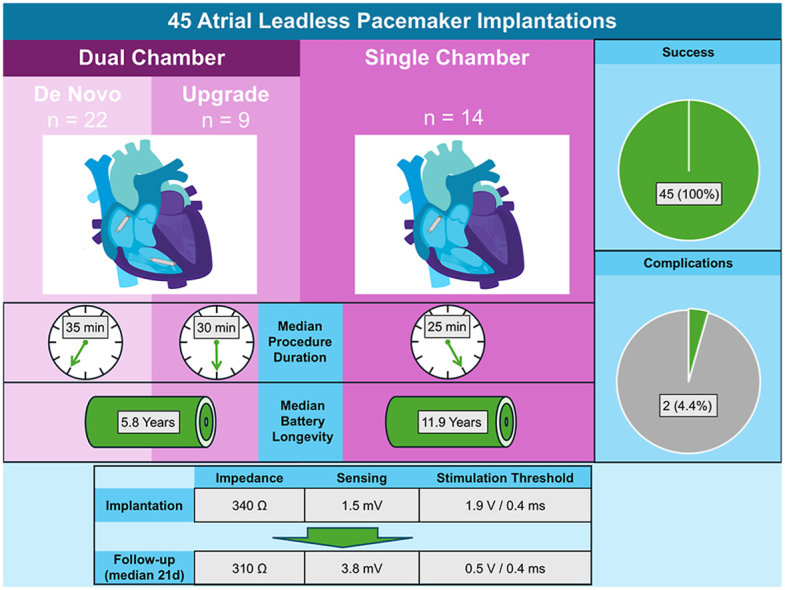

**Key Findings**

▪ In this first real-world European study, an atrial leadless pacemaker was implanted successfully in 45 patients without device repositioning or dislocation.
▪ Complications occurred in 4% of implantations (2 pericardial effusions without any needed interventions).
▪ The atrial leadless pacemaker demonstrated favorable device measurements at follow-up.
▪ Battery longevity and atrioventricular synchrony remain challenges during follow-up in case of leadless dual chamber pacemaker implantations.

## Introduction

Leadless pacemakers (LPs) have been associated with a lower incidence of acute and chronic complications compared with lead-based pacemakers (PM).^1^, ^2^, ^3^, ^4^ The first devices introduced in 2014 and 2016 were designed to be implanted in the right ventricle and were therefore only capable of pacing in VVI mode.^5^^,^^6^ The integration of a VDD mode using an accelerometer for mechanical atrial sensing in VDD mode improved atrioventricular (AV) synchrony to 70%–90%, depending on patient position, heart rate, and activity.^7^ However, the lack of leadless atrial pacing, insufficient AV synchrony at higher heart rates or during exercise, and the passive fixation that complicates device retrieval, all represented significant limitations for patient eligibility for LP implantation, as highlighted in recent guidelines.^8^ Subsequently, the world’s first active-fixation dual-chamber (DR) LP system was introduced (AVEIR DR; Abbott, Abbott Park, IL), allowing for atrial pacing, true electrical atrial sensing, and higher levels of AV synchrony facilitated by implant-to-implant (i2i) communication technology.^9^ Although device implantation and measurements demonstrated high success rates in the AVEIR DR LP (AVEIR, Abbott, Abbott Park, IL) Investigational Device Exemption (IDE) study, complications, including dislodgements and pericardial effusions, appeared more frequently, and implantation durations appeared to be higher compared with single chamber LP implantations. After the United States Food and Drug Administration approval in 2023 and CE mark in 2024, we present our initial prospective single-center European experience with our first patients implanted with an active fixation single chamber atrial device (AR) or DR LP system in the current study to evaluate its efficacy, safety, and efficiency in the real world.

## Methods

### Study design and patient population

This is a prospective descriptive single-center single-operator study. We included all patients from July 2024 until January 2025 who underwent implantation of an active fixation single chamber AR or DR LP and consented to study inclusion.

### Device description

**Figure 1:**
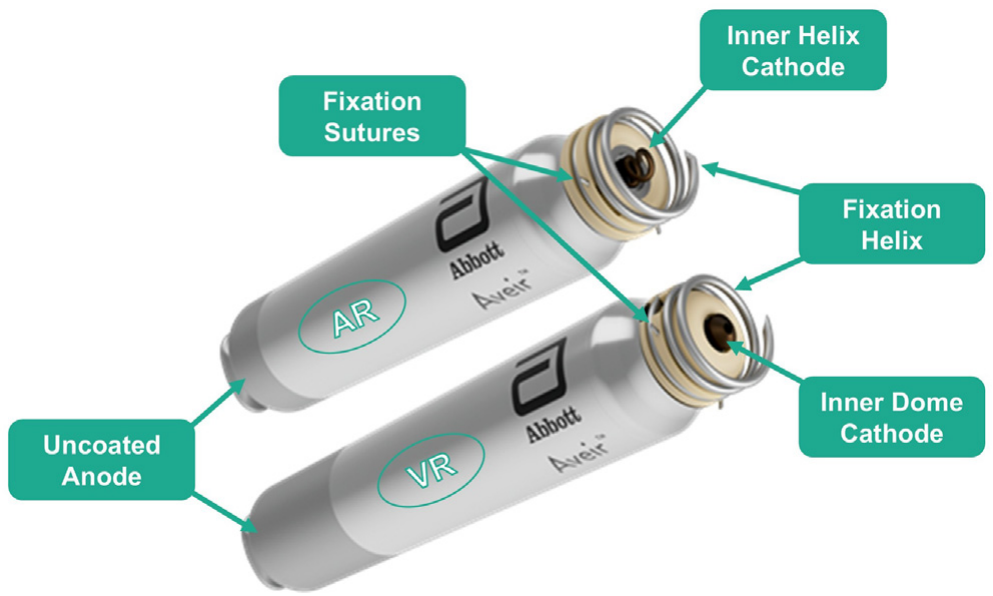
Atrial (AVEIR AR) and ventricular (AVEIR VR) leadless pacemaker. Both are 6.5 mm in diameter and feature an outer fixation helix with fixation sutures for torque-driven fixation and retrieval, an uncoated proximal anode, and a docking button. Although the VR features an inner dome cathode, the AR features a secondary inner helix as cathode and secondary fixation. They are both electrically isolated by a parylene coating. The AR is 32.2 mm, and the VR is 38 mm in length, respectively. AR = atrial device; VR = ventricular device.

The AVEIR DR LP system consists of an AR and ventricular device (VR) LP (Figure 1). Both devices integrate a pulse generator, battery, active fixation mechanism, and a docking interface. Their distal end features an external helix for primary active fixation. The inner, dome-shaped central electrode functions as the cathode in the VR, whereas a second smaller recessed helix acts as the cathode in the AR device. The proximal part of the LP case acts as the anode, with perylene insulation separating it from the cathode. Additionally, a docking button enables secure attachment and torque transmission during delivery and retrieval procedures. Although both LPs have a diameter of 6.5 mm, the AR is 32.2 mm, and the VR is 38 mm in length.

### Wireless PM synchronization

The AVEIR LPs can be used individually to feature atrial (AVEIR AR) or ventricular (AVEIR VR) pacing and sensing, or together in tandem to feature dual chamber pacing and sensing (AVEIR DR). Dual chamber pacing can be achieved through galvanic coupled intrabody communication, which was first evaluated in vivo on porcine hearts in 2018.^10^ The tissue (ie, myocardium and blood) is used as a transmission medium for electrical signals that are below the pacing threshold. The manufacturer-specific implementation of this technology is called i2i.^11^ The subthreshold signals are transmitted and received by the same electrodes and circuitry used for pacing. An i2i packet is sent before each pacing event and after each sensing event. It consists of a wake-up pulse (which activates a separate high-power receiver) and an actual i2i message containing 32 bits of information. The i2i packets initiate AV or VA intervals and blanking periods. They also contain information about the target pacing rate, magnet detection, battery status, and automatic mode switching. If no i2i packet is received, it is referred to as an i2i loss, which can occur in the AR-to-VR (A2V) direction, the VR-to-AR (V2A) direction, or both. However, safeguard modes are programmed to maintain synchronous pacing in case of i2i loss. For example, if DDD mode is programmed, an A2V i2i loss leads to a switch to DDI mode, a V2A i2i loss results in a switch to VDD mode, and a bidirectional loss triggers a switch to VDI mode. The devices also feature bridged pacing, allowing a singular atrial pacing event at the last rate interval in case of singular V2A i2i loss. The percentage of successful i2i communication is termed i2i throughput. To enhance successful i2i communication, the so-called i2i setting levels of the AR or VR device can be increased, which raises the amplitude of the transmitting signal and increases the receiving sensitivity, but also increases battery consumption.

### Implant procedure

**Figure 2:**
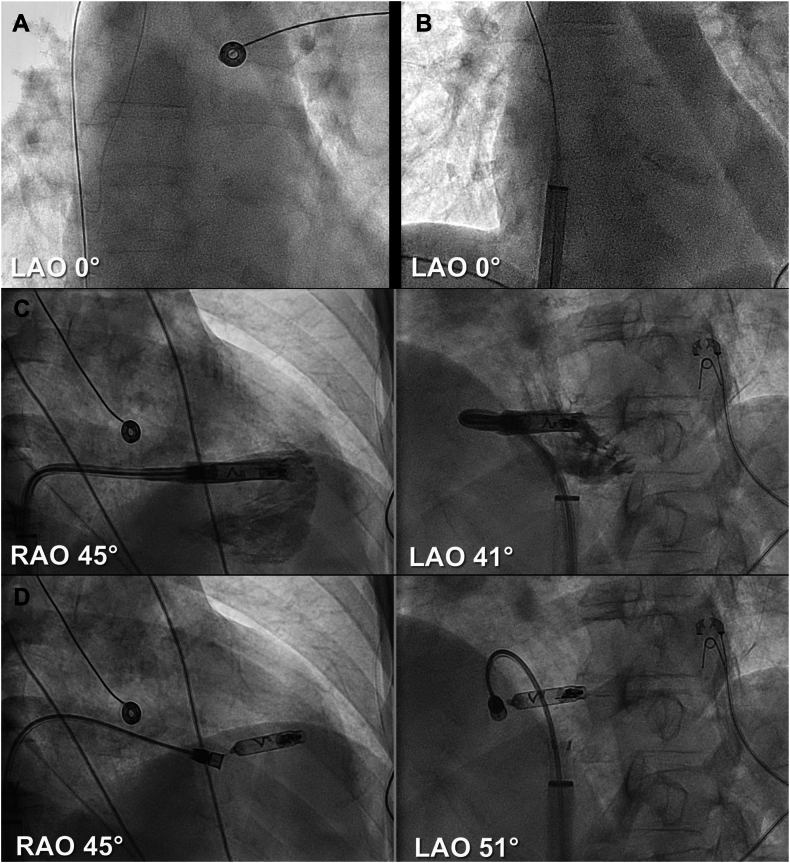
VR implantation. **A:** Stiff guidewire in vena cava superior. **B:** Delivery sheath in RA. **C:** Contrast-enhanced ventriculogram to facilitate optimal placement. **D:** Implanted AVEIR VR in the right ventricle in tether mode. LAO = left anterior oblique; RA = right atrial; RAO = right anterior oblique; VR = ventricular device.

**Figure 3:**
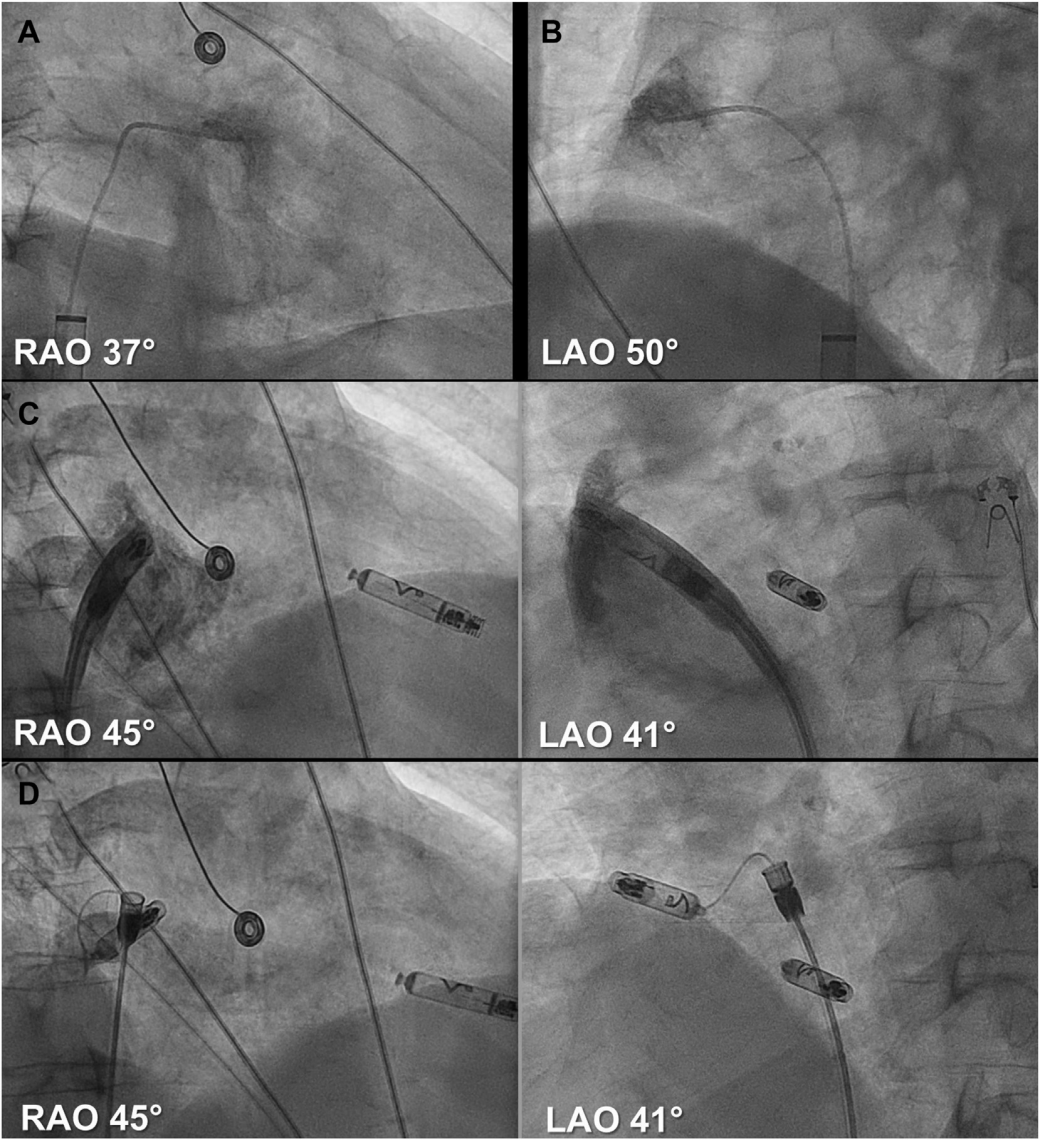
AR implantation. **A:** Contrast-enhanced visualization of the right atrial appendage through pigtail. **B:** Contrast-enhanced visualization of the right atrial appendage through protective sleeve over delivery catheter. **C:** Implanted AVEIR AR in the base of the right atrial appendage in tether mode. AR = atrial device; LAO = left anterior oblique; RAO = right anterior oblique.

All devices were implanted by 1 operator following previously reported guidelines by Neuzil and colleagues.^12^ A perioperative intravenous antibiotic prophylaxis was administered before the implantation. The procedure was performed under sterile conditions with local anesthesia at the femoral access site, and sedation was administered as needed (fentanyl/midazolam). Ultrasound-guided venous puncture was performed, and an introducer sheath was placed in the right atrium over a stiff guidewire to facilitate device delivery (Figures 2A and 3B). If a DR LP was implanted, the VR was implanted before AR. The VR was loaded onto the delivery catheter, advanced into the right atrium, and navigated across the tricuspid valve into the right ventricle in a preferably mid-septal position. Contrast injection was used to confirm septal placement and sufficient distance to the apex, the inferior groove, the anterior groove, and to the tricuspid valve (Figure 2C). Electrical testing was performed to assess pacing threshold, sensing, and impedance. Once optimal parameters were confirmed, the active helix fixation mechanism was deployed under fluoroscopic guidance. Stability was assessed using deflection maneuvers in tether mode to ensure secure fixation (Figure 2D). If device measurements were satisfactory, the device was released, and the delivery catheter was removed. During the first 15 AR implantations, a pigtail catheter was initially used for contrast injections delineating the anatomical structures in the right atrium (Figure 3A); thereafter, the contrast was administered through the delivery catheter itself (Figure 3B). The AR was loaded onto the delivery catheter, advanced into the right atrium, and positioned at the base of the right atrial appendage (RAA) under fluoroscopic guidance. Electrical testing was performed before the helix fixation mechanism was deployed, and stability was assessed using tension testing (Figure 3C). Once secure positioning and satisfactory device measurements were confirmed, the AR was released. With both devices released, final device measurements of the AR and VR devices were obtained.

### Follow-up and data acquisition

A device interrogation was performed in our hospital for all patients on the day of implantation, on the first day after implantation, and during a follow-up examination after 2–5 weeks. During this process, the impedance, pacing threshold, and P-wave sensing of the AR and, if applicable, R-wave sensing of the VR were recorded. In cases where an upgrade was performed, VR implantation values were recorded at the time of the VR implantation, but subsequent measurements (eg, at first day post-implantation and follow-up) were collected relative to the time of AR implantation. Additionally, during the follow-up examinations, the estimated battery life and the i2i throughput were documented. The i2i setting levels (Level 1–7) of both devices were adjusted according to the patient’s clinical situation, aiming at the lowest possible levels to provide clinically sufficient AV-synchrony.

### Statistical analysis

Continuous data are expressed as mean ± standard deviation or median and interquartile range (IQR) if not normally distributed. The Kruskal-Wallis test was used to compare non-parametric data. A *P*-value <0.05 was considered significant. The statistical analyses were performed using the statistical software R (version 4.4.1; R Foundation for Statistical Computing, Vienna, Austria) in RStudio (version 2024.12.0.467).

## Results

### Patient population

A total of 45 patients were implanted and included in our analysis. The mean age was 75.5 ± 10.9 years, the main indication was an atrioventricular block in 26 (57.8%), followed by sinus node disease in 15 patients (33.4%) (Table 1). A total of 22 patients (48.9%) received de novo DR implantation, 9 patients (20%) received an AR in addition to an already implanted VR (DR upgrade), and 14 patients (31.1%) received only an AR. In the patients with a DR upgrade strategy, the median time between the VR and AR implantation was 33 days (IQR 27–83).

**Table 1:** Baseline characteristics (all patients N = 45)

Age (y)	75.5 ± 10.9
Men	22 (48.9%)
BMI (kg/m^2^)	26.2 ± 4.6
Chronic kidney disease	16 (35.6%)
Hypertension	30 (66.7%)
Diabetes	9 (20.0%)
Dyslipidemia	21 (46.7%)
Atrial fibrillation	15 (33.3%)
Heart failure	6 (13.3%)
Coronary artery disease	6 (13.3%)
Valvular heart disease	4 (8.9%)
Malignancy	6 (13.3%)
Chronic obstructive pulmonary disease	2 (4.4%)
Obstructive sleep apnea syndrome	2 (4.4%)
Cerebrovascular disease	6 (13.3%)
Indications	
Sick sinus syndrome	15 (33%)
AV-block	26 (57.8%)
Binodal disease	3 (6.7%)
Pace-and-ablate	1 (2.2%)

The values given are mean or absolute numbers and standard deviations or percentages, respectively.

AV = atrioventricular; BMI = body mass index.

### Implant procedure and complications

All atrial devices were successfully placed at the base of the RAA, whereas ventricular devices (n = 31) were placed inferoseptal (22.5%), midseptal (67%), and anteroseptal (9.7%). No device repositioning was necessary. The median implantation time was 30 minutes (IQR 25–35), and the median fluoroscopy time was 2.8 minutes (IQR 1.5–3.9) (Table 2). Two patients (4.4%) experienced an implantation-related complication on the same day as the implantation (both experienced pericardial effusion). Both complications were managed conservatively and were self-limiting.

**Table 2:** Implantation characteristics (all patients N = 45)

Implantation time (min)	30 (IQR 25–35)
De novo DR (n = 22)	35 (IQR 30–40)
DR upgrade (n = 9)	30 (IQR 20–31)
AR only (n = 14)	25 (IQR 21–33)
Fluoroscopy time (min)	2.8 (IQR 1.5–3.9)
De novo DR (n = 22)	3.3 (IQR 2.3–4.8)
DR upgrade (n = 9)	1.6 (IQR 1–3.9)
AR only (n = 14)	2.7 (IQR 1.2–3.3)
Complications	2 (4.4%)
Pericardial effusion	2 (4.4%)
Dislocation	0
Other	0
AR position (n = 45)	
RAA base	45 (100%)
VR position (n = 31)	
Inferoseptal	7 (22.5%)
Midseptal	21 (67.7%)
Anteroseptal	3 (9.7%)

The values given are median or absolute numbers and interquartile range (IQR) or percentages, respectively.

AR = atrial device; DR = dual chamber system; RAA = right atrial appendage; VR = ventricular device.

### Device performance

**Figure 4:**
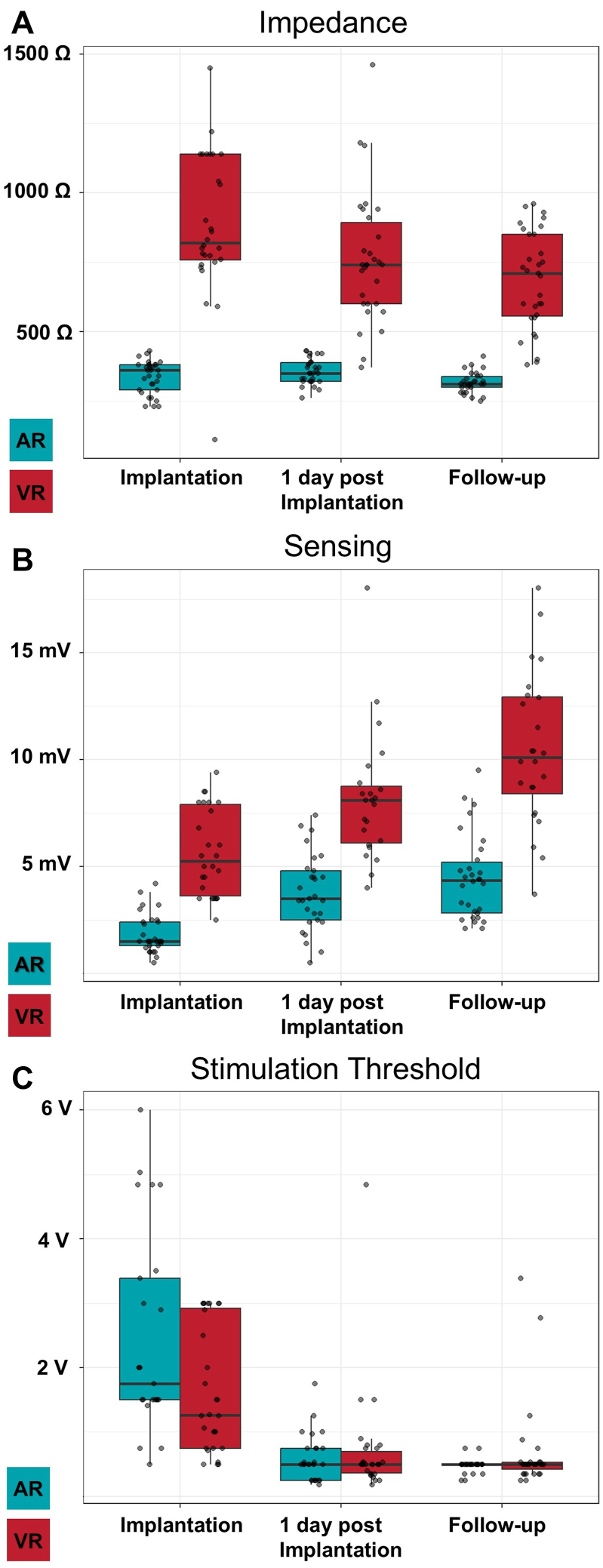
Device performance. **A:** Impedance, **B:** Sensing, and **C:** Threshold of the AVEIR AR and VR at implantation, 1 day post-implantation, and at follow-up. AR = atrial device; VR = ventricular device.

**Figure 5:**
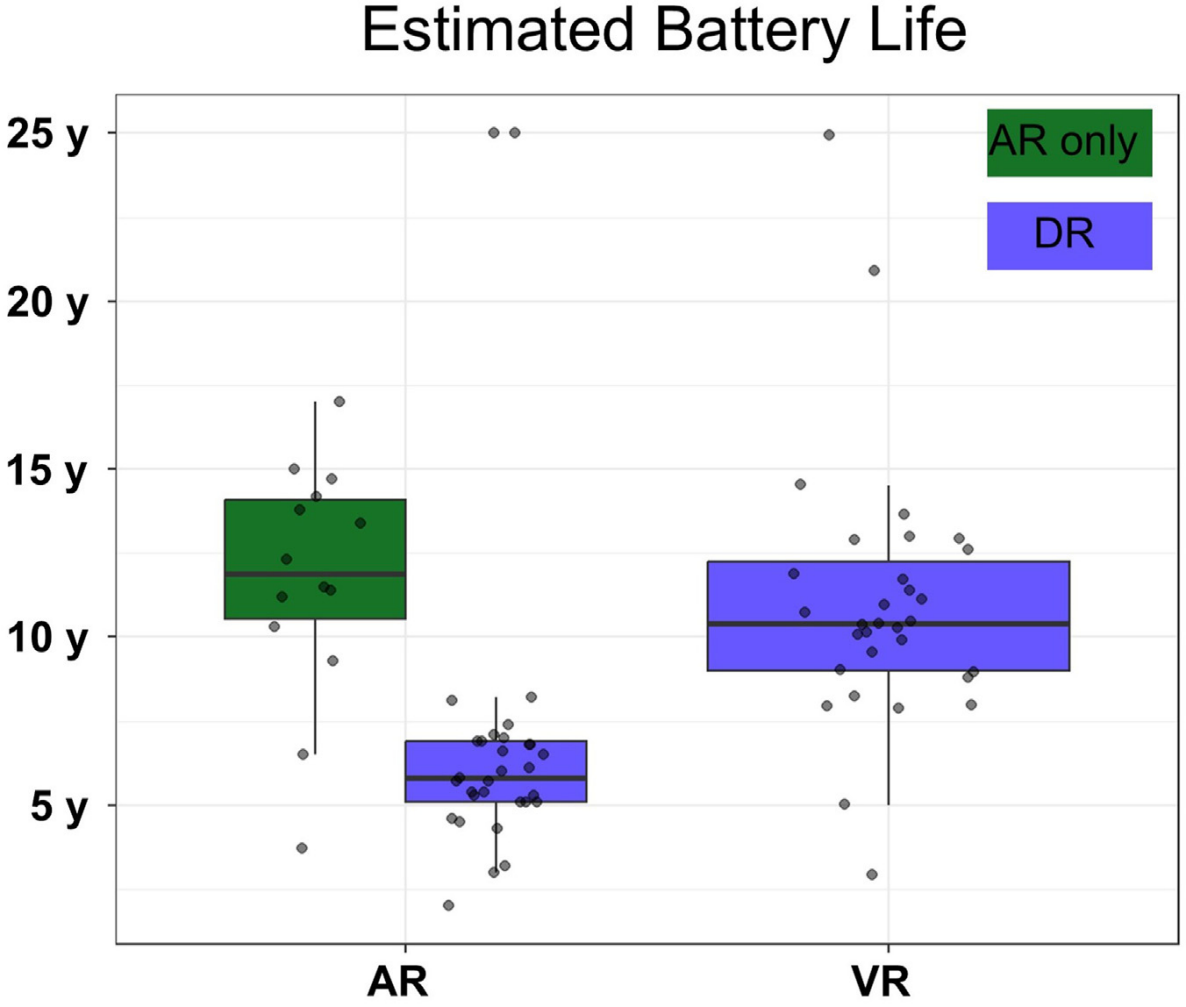
Estimated battery life at follow-up. The values were obtained at follow-up and are given in years (y). The values are separated by device (AR vs VR) and by pacing systems (AR only vs DR). AR = atrial device; DR = dual chamber system; VR = ventricular device.

**Figure 6:**
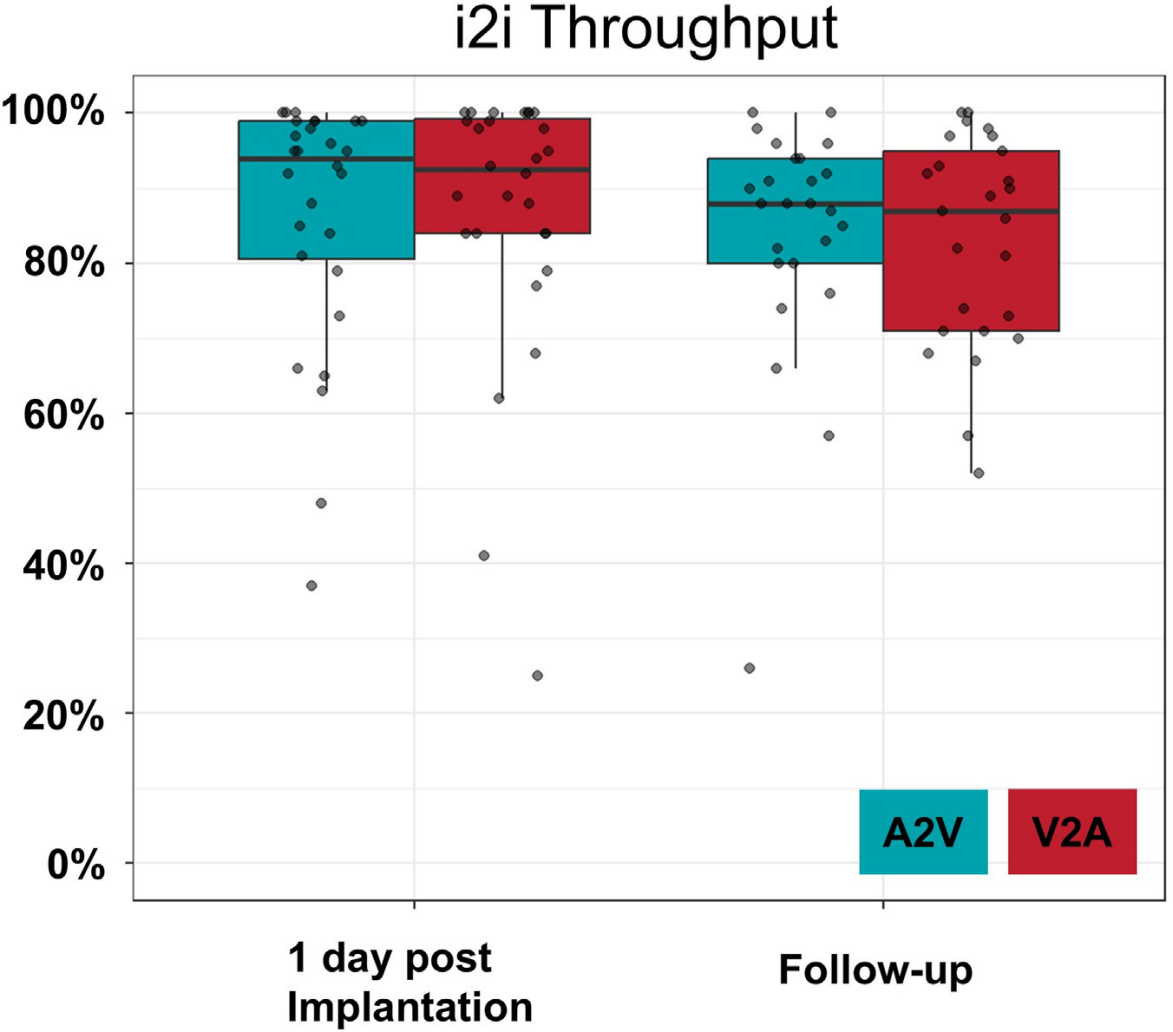
i2i throughput. Measurements of the percentage of received i2i packets by the ventricular device (A2V) and the atrial device (V2A) 1 day after implantation and at follow-up. A2V = AR-to-VR; AR = atrial device; V2A = VR-to-AR; VR = ventricular device.

The distribution of impedance, pacing threshold, and R-wave sensing of the atrial and ventricular devices is shown in Figure 4. The median follow-up time was 21 days (IQR 13–26). The median impedance was 340 Ω (IQR 290–380, n = 45) for AR and 820 Ω (IQR 757.5–1140, n = 28) for VR at implantation, which improved to 310 Ω (IQR 295–340, n = 44) for AR and 710 Ω (IQR 555–850, n = 31) for VR at follow-up. The median sensing was 1.5 mV (IQR 1.0–2.3, n = 44) for AR and 5.25 mV (IQR 3.6–7.9, n = 26) for VR at implantation, which improved to 3.75 mV (IQR 2.7–4.7, n = 44) for AR and 10.1 mV (IQR 8.4–12.9, n = 24) for VR at follow-up. The median pacing thresholds at implantation were 1.875 V/0.4 ms (IQR 1.5–3.47, n = 38) for AR and 1.26 V/0.4 ms (IQR 0.75–2.93, n = 28) for VR at implantation, which improved to 0.5 V/0.4 ms (IQR 0.5–0.5, n = 41) for AR and 0.5 V/0.4 ms (IQR 0.43–0.53, n = 31) for VR at follow-up. The median estimated battery life at follow-up of the AR was 6.8 years (IQR 5.3–11.2) and 10.4 years (IQR 9–12.2) of the VR. The median estimated AR battery life was significantly higher when implanted as an AR standalone compared to DR (11.9 years, IQR 10.5–14.1 vs 5.8 years, IQR 5.1–6.9, *P <* 0.001) as presented in Figure 5. Of the 31 patients with a DR system implanted, 2 device systems were set to a VVI mode. Therefore, a DDD mode and the usage of i2i communication were present in 29 patients. At follow-up, a median A2V-i2i throughput of 88% (IQR 80–94) and V2A throughput of 87% (IQR 71–05) was achieved (Figure 6). In 12 patients (41%), i2i was <80% in either A2V (3 patients), V2A (7 patients), or both directions (2 patients). A change of the i2i level was performed at follow-up in 19 patients (65%), either of the AR (7 patients) or of the VR (3 patients), or of both AR and VR (9 patients). The median i2i setting level after follow-up was 5 (IQR 4–5) in the AR and 4 (IQR 4–5) in the VR.

## Discussion

Our prospective single-center single-operator study offers a first insight into implantation and short-term follow-up results of a European real-world population with an atrial LP system. In our study, AVEIR AR implantation was successful in all patients without the need for device repositioning, with a low number of complications (< 5%) and acceptable device measurements during follow-up.

### Implantation

Our AVEIR AR implantation success rate was 100% with no device repositioning and short procedure and fluoroscopy times (median 30 and 2.8 minutes, respectively), whereas in the IDE study a similarly high implantation success rate of 98% but a higher rate of intraprocedural device repositioning (24%) and longer procedure and fluoroscopy times (mean 86.3 and 18.3 minutes, respectively) were observed.^9^ Explanations include the single operator design contributing to higher procedural consistency and efficacy in our study, post-IDE guidance on implantation style, target areas, and device stability tests, and post-IDE learnings of improving device measurements and i2i in the weeks after implantation.^13^ Our shorter procedure and fluoroscopy times and lower device repositioning rate may therefore represent the real-world implantation circumstances, incorporating the learnings since the IDE study.

### Safety

We observed pericardial effusions as complications in 2 patients (4%) after LP implantation, both of which were successfully managed conservatively. We observed no dislodgements of LP. In comparison, short-term complications were also observed in 4.2% of cases with lead-based PM and in 2% of cases with tine-fixed single chamber right ventricular LP.^1^^,^^14^ The IDE study reported a short-term complication rate of 9.7% with a pericardial effusion rate of 0.3% and a device dislodgement rate of 3.4%.^9^ The higher rate of pericardial effusion and lower rate of device dislodgement in our study may be explained by a lower patient sample size, 100% implantation location at the RAA base compared to 61% in the IDE study, or alternatively, by an operator preference difference in compromising stability vs perforation during the implantation.

### Device performance

Device measurements were favorable in our study: Stimulation thresholds of the LP were low and decreased over the first weeks, while the sensing improved, consistent with prior studies.^15^ The estimated battery longevity differed significantly from AR standalone to DR LP systems (11.9 years vs 5.8 years, *P <* .001), because of increased battery consumption due to i2i communication in a DR LP system. Battery longevity outliers represented 25 years in a DR system programmed to VVI because of only intermittent AV-block and < 4 years in patients with transiently high i2i setting levels or atrial output (Figure 5), underlining the importance of patient selection, regular device optimization to minimize battery consumption, and potential future enhancements of this technology (ie, AAI-DDD algorithms, reduced i2i battery consumption, increased atrial battery size, increased atrial impedance). We achieved acceptable i2i throughputs (A2V 88% [IQR 80–94], V2A 87% [IQR 71–95]) in the follow-up period with median i2i setting levels of 5 (A2V) and 4 (V2A). However, these were lower than achieved in the IDE study (A2V 93.6%, V2A 94.1%).^9^ Potential explanations include differences in patient population (ie, our study included 100% AV-block in DR LP systems vs IDE study included 33% AV-block patients), a shorter follow-up time (2 weeks in our study vs 3 months in the IDE study), and physician differences in targeted i2i throughput. It is important to realize, that i2i throughput tends to mark the lower boundary of AV-synchrony because of safeguard algorithms (see: Methods, Wireless PM Synchronization) and competitive factors influencing i2i (premature ventricular contractions, atrial fibrillation, etc.), therefore an i2i throughput of 88% may facilitate an AV-synchrony > 90%.^13^^,^^16^ An optimization of the i2i level was performed in most patients during our early follow-up visits (61%), therefore, a longer follow-up is required to assess the long-term i2i communication and AV-synchrony of these devices.

### Limitations

This is a single-center and single-operator study with a limited sample size and follow-up time, and has all the inherent limitations of this study design. Because the follow-up time was limited, we did not report any data regarding the effects of non-physiologic right ventricular pacing in our cohort. The scarce data on long-term performance and retrievability should be discussed with the patients and weighed in the difficult evaluation of LP vs lead-based PM implantation.

## Conclusion

Our first real-world European experience demonstrates that leadless atrial PM implantation with active fixation can be performed efficiently and safely, with satisfactory device measurements during an early follow-up period. However, patient selection, evaluation of staged or primary dual chamber implantation, and regular follow-ups, including device optimizations, are needed to optimize device performance, especially concerning i2i communication and battery consumption.
